# Amazon Employees Resources Access Data Extraction via Clonal Selection Algorithm and Logic Mining Approach

**DOI:** 10.3390/e22060596

**Published:** 2020-05-27

**Authors:** Nur Ezlin Zamri, Mohd. Asyraf Mansor, Mohd Shareduwan Mohd Kasihmuddin, Alyaa Alway, Siti Zulaikha Mohd Jamaludin, Shehab Abdulhabib Alzaeemi

**Affiliations:** 1 School of Distance Education, Universiti Sains Malaysia, Penang 11800, Malaysia; ezlinzamri@student.usm.my (N.E.Z.); alyaalway@student.usm.my (A.A.); 2 School of Mathematical Sciences, Universiti Sains Malaysia, Penang 11800, Malaysia; shareduwan@usm.my (M.S.M.K.); szulaikha.szmj@usm.my (S.Z.M.J.); shehab_alzaeemi@yahoo.com (S.A.A.)

**Keywords:** Boolean satisfiability, clonal selection algorithm, data extraction, human resources management, logic mining

## Abstract

Amazon.com Inc. seeks alternative ways to improve manual transactions system of granting employees resources access in the field of data science. The work constructs a modified Artificial Neural Network (ANN) by incorporating a Discrete Hopfield Neural Network (DHNN) and Clonal Selection Algorithm (CSA) with 3-Satisfiability (3-SAT) logic to initiate an Artificial Intelligence (AI) model that executes optimization tasks for industrial data. The selection of 3-SAT logic is vital in data mining to represent entries of Amazon Employees Resources Access (AERA) via information theory. The proposed model employs CSA to improve the learning phase of DHNN by capitalizing features of CSA such as hypermutation and cloning process. This resulting the formation of the proposed model, as an alternative machine learning model to identify factors that should be prioritized in the approval of employees resources applications. Subsequently, reverse analysis method (SATRA) is integrated into our proposed model to extract the relationship of AERA entries based on logical representation. The study will be presented by implementing simulated, benchmark and AERA data sets with multiple performance evaluation metrics. Based on the findings, the proposed model outperformed the other existing methods in AERA data extraction.

## 1. Introduction

Amazon.com Inc. operates internationally by offering consumers products and subscriptions through more than 10 owned retail websites and physical stores in 600 locations across the United States of America (US). As reported in 2019, the company has increasing numbers of employees, and more than 600,000 employees worldwide [1]. Thus, with a large number of employees, there is always a risk of highly complicated employees and resources situations [2]. Within any company, new employees require a variety access of systems, portals or appliances related on the role, designation or unit of the employee. Technology companies like Amazon.com Inc. provide various types of resources; from computing to storage resources, accessible by their employees to be utilized optimally [3]. However, most of the time, employees encounter some complications prior to fulfilling their daily tasks. For example, computing resources opt to have Wi-Fi connection or they are unable to log in into Amazon.com Inc. human resources portal. Commonly, new resources applications are being processed and reviewed by distinct human administrators. It is worth mentioning that the downside of this common practice involves a chain of human involvement that could lead to higher cost of resource maintenance and could be time-consuming. Therefore, Amazon.com Inc. made public their historical data from 2010–2011 of Amazon Employees Resources Access (AERA) data set, provisioned by Ken Montanez from Information Security of Amazon.com Inc. in partnership with Kaggle. Their motive is to seek alternative models that will prioritize the needs of employees and minimize manual resources access applications. A study by [4] proposed a forecasting model by using random forest (RF), logistic regression (LR) and gradient boosting (GB). However, the suggested approach was restricted to statistical linear classifiers and required a preprocessing step due to the imbalanced entries of AERA. One may question what makes this experiment significant from the work by [4]? In this paper, the main objective is to propose an alternative model in the field of data science by incorporating Artificial Neural Networks (ANNs) with Metaheuristic and Satisfiability representation (SAT). The proposed model act as a platform of knowledge extraction to handle big data which could benefit other big companies like Walmart Inc., Apple Inc., Samsung Electronics etc. in resources management.

ANN comprises parallel and nonparallel computing networks that are inspired by the mechanism of human biological brain [5]. ANN has several comprehensive architectures of feed-forward or feedback networks. Artificial Intelligence (AI) practitioners utilized ANN as a platform in applications such as entity classification problems [6], conducting analysis [7,8], pattern recognition [9,10], clustering problems [11,12] and circuits [13,14]. Nonetheless, another popular network of feedback ANN is the Hopfield Neural Network (HNN), which was formulated by [15] to solve optimization tasks. The extensive structure of HNN comprises energy function and associative property of content addressable memory (CAM). The work of [16] utilized HNN for transmitting binary amplitude modulated signals based on the potential energy function yielding lower probability of error. In addition, the work of [17] emphasized HNN as one of the most studied attractor-memory models due to the feature of useful Content Addressable Memory (CAM) for an optimization model. Note that HNN can be split into continuous HNN (CHNN) and discrete HNN (DHNN). The structure of DHNN consists of input and output neurons that store bipolar {1,−1} or binary {1,0} pattern [18]. In addition, DHNN utilizes the Lyapunov energy function to determine degree of convergence of the solution [19]. This paper incorporates the Wan Abdullah (WA) method of finding the synaptic weights by comparing the Lyapunov energy function with the cost function [20]. The core impetus of the presented works is the relevancy of utilizing DHNN as a comprehensive model of AI as a platform to solve optimization tasks. Although DHNN is a “black box” model, the best way to observe DHNN behaviour is by implementing a systematic symbolic rule during the learning phase and a retrieved phase equation. Hence, one of the alternative ways to represent information theory is by the concept of satisfiability.

Satisfiability representation (SAT) is a logical and mathematical knowledge representation that plays a significant role in AI. SAT is utilized in various applications and areas such as quantum chemistry [21], approximation model [22], classification [23], chaos computing [24] and fault detection [25]. The SAT structure consists of clauses comprises of literals or variables. Why is SAT needed in DHNN? SAT is essential to provide symbolic instruction in attempt to represent the output of DHNN. Pioneer work by [26] showed the adaptability of Horn-SAT to represent information in executing the DHNN model that was improved later by [27]. The work improvised the existing model in neuro-symbolic integration model that gained more than 90% of global minimum energy. However, the restricted component in using Horn formula is limited in representing real-life data sets, which indicate not all real-life problem can be formulated in Horn-SAT [28]. Therefore, several researchers further extend the fundamental of Horn-SAT by proposing of DHNN model with different *k*-Satisfiability (*k*-SAT) logical representation [29,30,31]. These works emphasized on utilizing *k*-SAT, Maximum *k*-SAT (MAX*k*-SAT) and Maximum 2-SAT (MAX2-SAT) to investigate the ability of DHNN to process *k*-SAT patterns. In another development, data mining is a process of recognizing sequences or patterns in real-life data sets that involve various platforms. The difference between data mining and logic mining is that the logical rule mining utilizes logic to convey the information to the end user. Contingent upon that, the earliest logic mining method, the reverse analysis (RA) method, was introduced by [32] and it accommodates the combination of RA and logic programming in DHNN to deduce the pattern and relationship of the real-life data sets. Subsequently, [33] utilized previous work of building a knowledge extraction tool by forming *k*-Satisfiability-based Reverse Analysis (*k*-SATRA). *k*-SATRA carries an important role in logic mining to display the true behaviour or pattern of a real-life data set by extracting the optimum logic that represents the relationship of the attributes. The extracted logic will represent information aligned with the specifics classification tasks. An interesting application of *k*-SATRA is reported by the work of [34], which investigates students’ performance in identifying related factors of underachievement students. The work entrenched several real-life data sets and obtained higher accuracy than two other existing educational data mining methods. Another development of utilizing *k*-SATRA was by [35] and [36] which exhibited the ability 2-SATRA to extract key findings of online games and football matches. The common denominator of these works exhibits the practicability of *k*-SATRA in extracting knowledge from a real-life data set. The extracted knowledge identifies relationships of attributes that affect the final outcome. However, there are no current works creating a platform to bridge logic and data mining methods with specific optimization tasks such as those encountered by Amazon.com Inc. of detecting which factor should be prioritized in order to grant or revoke employees resources applications. The incorporation of metaheuristics like the Clonal Selection Algorithm (CSA) in the training phase would capitalize better on the learning environment for an optimal optimization model.

The Metaheuristics Algorithm is a nonderivative method that searches near optimal solutions with specific constraints. [37] presented various applications of metaheuristics to find high-quality solutions to increasing number of ill-defined and complex real-world problems. Metaheuristics garnered much attention, especially from ANN practitioners, because metaheuristics provides a better learning mechanism of ANN networks by specifying the searching space of solutions and focusing on gradual solution improvement [38]. Conventionally, DHNN deployed the primitive learning rule of exhaustive search (ES), a trial and error mechanism to find solutions [39]. ES increases the probability of overfitting [40] and generates less variation of solutions [41]. CSA is an evolutionary algorithm, inspired by the natural phenomenon of the biological immune system, which defends the body against external microorganisms. [42] reviewed recent works by researchers implementing CSA into their proposed network to deal with constraint optimization tasks, such as pattern recognition [43], scheduling [44], fault detection [45] and dynamic optimization [46]. Mechanisms of CSA gives the inspiration of specific cells to recognize specific antigens which are later selected to proliferate. This resulted in a learning algorithm of evolving candidate solutions by selection, cloning and somatic hypermutation procedures, which established variation of solutions. Conjointly, the mechanism of CSA sets a new paradigm of solving optimization tasks. Pioneer work by [47] introduced the affinity-based interaction for modified CSA as a solver with the tabu search technique for the Maximum 3-SAT (MAX3-SAT) problem. The suggested model yielded quality solutions. Therefore, to predict the resources access applications for future sets of employees of AERA, this paper capitalizes on fundamental DHNN by incorporating CSA in the learning phase to overcome conventional metaheuristic drawbacks. The proposed model sets apart from previous literature due to different role of CSA to facilitate the learning phase of DHNN for 3-SAT logic, resulting a single intelligent unit that incorporates real-life data set to help Amazon.com Inc. resources access management.

**Figure 1:**
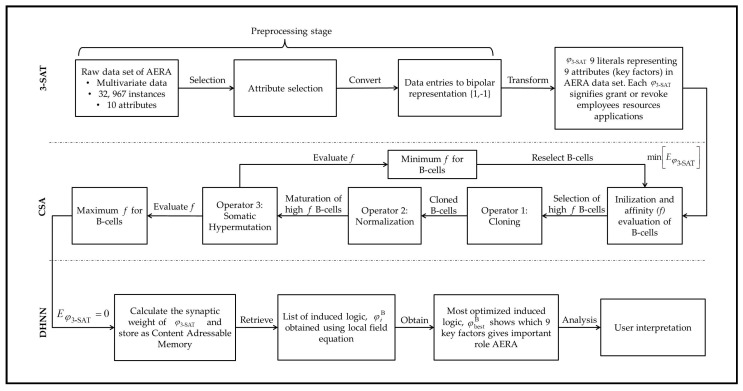
Implementation of the proposed model.

To our best knowledge, no current work has proposed the incorporation of DHNN with CSA for 3-SATRA logic-mining methods. An optimal model may result in better management from Amazon.com Inc. in providing the best care for their employees. Subsequently, the contributions of this work are stated as follows: (1) To transform AERA into a 3-SAT logical representation to best represent the relationship of AERA. (2) To construct a modified DHNN model with CSA to enhance the learning phase of DHNN. (3) To utilize the 3-SATRA method into our proposed model as an alternative method to extract information from AERA in the form of logical representation. (4) To demonstrate the capability of our proposed model by conducting a simulated data set, benchmark data sets and the AERA data set in comparison with other existing methods. The comparison will be also evaluated by using appropriate performance evaluation metrics. The findings of this paper displayed the competency of our proposed model outperformed other existing methods for all type of data sets. Figure 1 illustrates the implementation of our contribution in this paper.

## 2. Boolean Satisfiability

Boolean satisfiability logic (SAT) represents a task in determining truth assignments that makes the logical rule satisfiable. SAT is a nondeterministic polynomial time, NP-complete problem where SAT can be solved in polynomial time by a nondeterministics Turing machine [48]. In this paper, SAT is represented in a conjunctive normal form (CNF) and composed of three significant elements [49]:

A group of m variables: $a_{1}\vee a_{2}\vee \ldots \vee a_{m}$ where $a_{i}\in \left\{1,-1\right\}$.
A group of literals: A literal is a variable $\left(a_{1}\right)$ or a negation of a variable $\left({\bar{a}}_{1}\right)$.
A group of n clauses: $A_{1}\wedge A_{2}\wedge \ldots \wedge A_{n}$.

The above elements can be explicitly represented in the following Equation (1):

(1) $$\varphi_{3-\mathrm{SAT}}=\overset{n}{\underset{i=1}{\wedge }}A_{i}\;\mathrm{where}\;A_{i}=(a_{i},b_{i},c_{i})$$

This paper utilized 3-Satisfiability (3-SAT) logical rule, $\varphi_{3-\mathrm{SAT}}$, in each clause of which only exist three variables. Equation (2) governed an example of $\varphi_{3-\mathrm{SAT}}$. Note that $\varphi_{3-\mathrm{SAT}}$ represents the objective or outcome of the logical rule.

(2) $$\varphi_{3-\mathrm{SAT}}=\left(\bar{P}\vee Q\vee R\right)\wedge \left(S\vee \bar{T}\vee \bar{U}\right)\wedge \left(\bar{V}\vee W\vee X\right)$$

Table 1 shows an example of cases for the $\varphi_{3-\mathrm{SAT}}$ logical rule. The outcomes of each case are known by substituting the values of {1,−1} (neuron states) into Equation (2). For instance, case 1 is satisfiable since each clause gives a truth value. Besides that, case 3 is in full consistency since all literals give a truth value. Additionally, the work by [49] states that the algorithm needs to learn more inconsistent interpretations to obtain the satisfied $\varphi_{3-\mathrm{SAT}}$, which we described as the checking clause satisfaction process. To undergo this process, a suitable metaheuristic algorithm is needed to attain $\varphi_{3-\mathrm{SAT}}=1$ [47]. In this paper, the $\varphi_{3-\mathrm{SAT}}$ logical rule is employed in our proposed model to govern our model and represent each entry of AERA.

## 3. 3-Satisfiability in Discrete Hopfield Neural Network

The Discrete Hopfield Neural Network (DHNN) is another variant of the Hopfield Neural Network that is commonly utilized to solve practical optimization problems [50]. DHNN consists of interconnected neurons with no hidden layer. Each neuron in DHNN is bipolar $S_{i}\in \left\{-1,1\right\}$, i∈ℕ, which exemplifies the interpretation of the defined problem. Several properties of DHNN include associative memory, fault tolerance and energy minimization as the neuron state changes. There are two types of neuron updates in DHNN: asynchronous and synchronous update. We limit our discussion to asynchronous update because we only consider one neuron state at the time. Each neuron spin resembles an Ising spin variable model [51], which contributes to updating neurons in each cycle. The general updating rule of the general DHNN is given as follows:

(3) $$S_{i}=\left\{ \begin{array}{l} \;1,\;\mathrm{if}\;\sum_{j}^{N}W_{ij}S_{j}\geq \rho_{i}\\ -1\;,\;\mathrm{otherwise} \end{array}\right.$$

where $W_{ij}$ and $\rho_{i}$ are the synaptic weight and threshold of the contraints. It is worth mentioning that we consider $\rho_{i}=0$ to ensure the energy of DHNN decreases uniformly [52]. $W_{ij}$ in each neurons connection is formally defined in a matrix of $W_{ij}^{\left(2\right)}={\left[W_{ij}^{\left(2\right)}\right]}_{N\times N}$ with the threshold of the neuron updates given by ${\left[\rho_{i}\right]}_{n\;\times \;1}={\left[\rho_{1},\rho_{2},\rho_{3},\ldots ,\rho_{n}\right]}^{T}$. Note that DHNN has no self-looping $W_{ii}^{\left(2\right)}=W_{jj}^{\left(2\right)}=0$ for all neurons and the connection is symmetrical $W_{ij}^{\left(2\right)}=W_{ji}^{\left(2\right)}$ which results in a matrix with zeros diagonal. The updating rule of general DHNN is important to ensure the neuron state will converge to the optimal solution. In this section, we capitalize the logical rule of $\varphi_{3-\mathrm{SAT}}$ into the structure of DHNN by defining the cost function of the network. $\varphi_{3-\mathrm{SAT}}$ can be implemented in DHNN by minimizing the cost function $E_{\varphi_{3-\mathrm{SAT}}}$:

(4) $$E_{\varphi_{3-\mathrm{SAT}}}=\sum_{i=1}^{NC}\prod_{j=1}^{N}D_{ij}$$

where NC and N are the number of clauses and number of literals accordingly. $D_{ij}$ is defined as follows:

(5) $$D_{ij}=\left\{ \begin{array}{l} \frac{1}{2}\left(1+S_{X}\right)\;,\;\mathrm{if}\;X\neq \bar{X}\\ \frac{1}{2}\left(1-S_{X}\right)\;,\;\mathrm{otherwise} \end{array}\right.$$

where X is one possible variable in $\varphi_{3-\mathrm{SAT}}$. Note that the lowest value of the cost function is $E_{\varphi_{3-\mathrm{SAT}}}=0$ where all the inconsistencies of the $\varphi_{3-\mathrm{SAT}}$ are minimized. Hence the updating rule or local field of the $\varphi_{3-\mathrm{SAT}}$ in DHNN is given as follows:

(6) $$h_{i}\left(t\right)=\sum_{k=1,k\neq j}^{N}\sum_{j=1,j\neq k}^{N}W_{ijk}^{\left(3\right)}S_{k}S_{j}+\sum_{j=1,j\neq \;i}^{N}W_{ij}^{\left(2\right)}S_{j}+W_{i}^{\left(1\right)}$$

(7) $$S_{i}\left(t+1\right)=\left\{ \begin{array}{l} 1\;,\;\sum_{k=1,k\neq j}^{N}\sum_{j=1,j\neq k}^{N}W_{ijk}^{\left(3\right)}S_{k}S_{j}+\sum_{j=1,j\neq \;i}^{N}W_{ij}^{\left(2\right)}S_{j}+W_{i}^{\left(1\right)}\geq 0\\ -1\;,\;\sum_{k=1,k\neq j}^{N}\sum_{j=1,j\neq k}^{N}W_{ijk}^{\left(3\right)}S_{k}S_{j}+\sum_{j=1,j\neq \;i}^{N}W_{ij}^{\;(2)}S_{j}+W_{i}^{\left(1\right)}<0 \end{array}\right.$$

where $W_{ijk}^{\left(3\right)}$, $W_{ij}^{\left(2\right)}$ and $W_{i}^{\left(1\right)}$ are synaptic weight for the third, second and first order connection respectively. The threshold for the proposed DHNN is ρ=0 and can be flexibly defined by the user. According to [53], the final neuron state, $S_{i}\left(t+1\right)$, can be optimized by the usage of a squashing function such as a Hyperbolic Activation Function (HTAF). Interested readers on this aspect may refer to [49,53,54]. Furthermore, Equations (6) and (7) are vital to ensure the final neuron state always converges to $E_{\varphi_{3-\mathrm{SAT}}}\to 0$. Theorem 1 explains the behaviour of the synaptic weight with respect to the final state of the neuron.

**Theorem** **1.** *Let* N=(W, ρ) *where* ρ *is the threshold of the model of DHNN. Assuming* N *operates in asynchronous mode and* W *is a symmetric matrix with the elements of the diagonal being nonnegative. Then DHNN will always converge to a stable state.*

In addition, the Lyapunov energy function $L_{\varphi_{3-\mathrm{SAT}}}$ that corresponds to the $\varphi_{3-\mathrm{SAT}}$ rule is given as follows:

(8) $$L_{\varphi_{3-\mathrm{SAT}}}=-\frac{1}{3}\sum_{i=1,i\neq j\neq k}^{N}\sum_{j=1,i\neq j\neq k}^{N}\sum_{k=1,i\neq j\neq k}^{N}W_{ijk}^{\left(3\right)}S_{i}S_{j}S_{k}-\frac{1}{2}\sum_{i=1,i\neq j}^{N}\sum_{j=1,i\neq j}^{N}W_{ij}^{\left(2\right)}S_{i}S_{j}-\sum_{i=1}^{N}W_{i}^{\left(1\right)}S_{i}$$

The value of $L_{\varphi_{3-\mathrm{SAT}}}$ indicates the quality of the final state obtained from Equation (8). According to [20], the synaptic weight of the DHNN can be obtained by comparing Equations (4) and (6). The energy value of the $\varphi_{3-\mathrm{SAT}}$, $L_{\varphi_{3-\mathrm{SAT}}}^{\min}$, can be predetermined before the learning phase because the energy value from each clause in $\varphi_{3-\mathrm{SAT}}$ is always constant. It is worth mentioning that the optimal DHNN always converges to $L_{\varphi_{3-\mathrm{SAT}}}\to L_{\varphi_{3-\mathrm{SAT}}}^{\min}$ or $\left|L_{\varphi_{3-\mathrm{SAT}}}-L_{\varphi_{3-\mathrm{SAT}}}^{\min}\right|\leq \partial$, where ∂ is the tolerance value of the Lyapunov energy function. In this paper, the information from the data set will be represented in terms of $\varphi_{3-\mathrm{SAT}}$ and embedded into DHNN. The implementation of $\varphi_{3-\mathrm{SAT}}$ in DHNN is abbreviated as DHNN-3SAT. One of the main obstacles in implementing DHNN-3SAT is to find a set of $S_{i}$ that corresponds to $E_{\varphi_{3-\mathrm{SAT}}}=0$. By that standard, optimal learning method is required to effectively minimize $E_{\varphi_{3-\mathrm{SAT}}}$.

## 4. Clonal Selection Algorithm

The learning phase of an ANN can be further improved via metaheuristics to provide more global solutions, a better learning mechanism and to ascertain the convergence of the ANN models [55]. A work proposed by [56] indicated that these algorithms required less execution time to complete the training process. Generally, metaheuristics have two types of searching algorithms, trajectory-based and population-based. The work is focusing on the population-based nature-inspired algorithm of evolutionary algorithms (EA). CSA is a class of Artificial Immune System (AIS) algorithms that is motivated by the natural immune system process that build particular antibodies against antigens. B-cells (β) will produce specific antibodies once a new antigen is identified. Through the cloning process, the chosen β will proliferate to form a clone of β and fight against antigens [57]. The cloned β developed into two types of cell, memory cells and plasma cells. Memory cells are recognized as long-lived cells that can react instantly to any illness. As for the plasma cells, they are active and able to secrete specific antibodies for the antigens, but they do not last long.

The findings by Layeb [47] presented modified CSA with the tabu search method to resolve the satisfiability problem. The affinity computation in [47] utilized the adaptive affinity function, which considers the summation of weight with the clauses and complies with the binary vector form of MAX-SAT logical representation. On the other hand, our proposed CSA model complies to bipolar representation of $\varphi_{3-\mathrm{SAT}}$ as the affinity function being formulated in terms of clause representation that corresponds to $E_{\varphi_{3-\mathrm{SAT}}}=0$. The operations involved in the CSA mechanism are where β produces a specific antibody to destroy a specific antigen, which signifies the adaptive system of CSA principle. Proliferation, normalization and somatic hypermutation processes ensure a better variation of the β population. This paper implements the CSA mechanism to provide an optimal learning model, where CSA helps to achieve maximum number of satisfied clauses from the affinity or fitness of β. The implementation of CSA in the proposed model (DHNN3-SATCSA) is presented as follows [58]:

**Stage 1:** Initialization of β

β=100 (interpretations) were initialized.

(9) $$\beta_{ij}=\left\{ \begin{array}{cc} 1\;, & rand\left[0,1\right]\geq 0.5\\ -1\;, & \mathrm{otherwise} \end{array}\right.$$

**Stage 2:** Affinity Evaluation

Compute affinity of all β in the entire population, $f_{\beta_{i}}$. The $f_{\beta_{i}}$ examines the number of clauses satisfied in $\varphi_{3-\mathrm{SAT}}$. $A_{i}$ is the number of clauses learned by CSA and NC is the number of clauses in $\varphi_{3-\mathrm{SAT}}$.

(10) $$f_{\beta_{i}}=\sum_{i=1}^{NC}A_{i}$$

where

(11) $$A_{i}=\left\{ \begin{array}{l} \;1\;,\;True\\ \;0\;,\;False \end{array}\right.$$

given that $f_{\beta_{i}}\in \mathbb{Z}$.

**Stage 3:** Proliferation via Cloning

The top five β with higher affinity were chosen to proliferate in cloning process. In this process, β will be duplicated by applying the roulette wheel mechanism [59]. The number of cloned β, $N_{\varepsilon }$ will be computed by using Equation (12).

(12) $$N_{\varepsilon }=\frac{\lambda f_{i}}{\sum_{i=1}^{NC}f_{\beta_{i}}}$$

where λ, known as initial affinity, is the population clone size which the software seeks to implement into the searching space. [58] suggested selecting λ=200.

**Stage 4:** Normalization

Equation (13) shows the list of cloned β; $1\leq \beta_{i}^{C}\leq N_{\varepsilon }$.

(13) $$\beta_{i}^{C}=\left\{\beta_{1}^{C},\beta_{2}^{C},\beta_{3}^{C},\ldots ,\beta_{N_{\varepsilon }}^{C}\right\}$$

Normalization of $\beta_{i}^{C}$ $\left({\tilde{\beta }}_{i}^{C}\right)$ is often called immune response maturation throughout the system. It is important to normalize the $\beta_{i}^{C}$ before proceeding to the next step. Next, we calculate the affinity for each ${\tilde{\beta }}_{i}^{C}$, which is abbreviated as $f_{{\tilde{\beta }}_{i}^{C}}$.

(14) $$f_{{\tilde{\beta }}_{i}^{C}}=\frac{f_{\beta_{i}^{C}}-\min\left|f_{\beta_{i}^{C}}\right|}{\max\left|f_{\beta_{i}^{C}}\right|-\min\left|f_{\beta_{i}^{C}}\right|}$$

where

(15) $$\max\left|f_{\beta_{i}^{C}}\right|-\min\left|f_{\beta_{i}^{C}}\right|>0$$

Note that $\max\left|f_{\beta_{i}^{C}}\right|\neq \min\left|f_{\beta_{i}^{C}}\right|$ because the probability of getting $f_{\beta_{i}^{C}}=0$ is almost zero.

**Stage 5:** Somatic Hypermutation

The somatic hypermutation process is significant since it will ensure the β to achieve highest affinity which results in a feasible solution. Equation (16) shows the calculation of the number of mutations $\left(N_{\zeta }\right)$ for each ${\tilde{\beta }}_{i}^{C}$.

(16) $$N_{\zeta }=\left(\frac{f_{{\tilde{\beta }}_{i}^{C}}}{\eta }\right)+\theta \left(1-f_{{\tilde{\beta }}_{i}^{C}}\right)$$

where η is the number of variables in $\varphi_{3-\mathrm{SAT}}$, θ=0.01 and η≠0 [58]. For every mutation that occurs in $N_{\zeta }$, one or more β will be flipped from 1 to −1 or vice versa.

**Figure 2:**
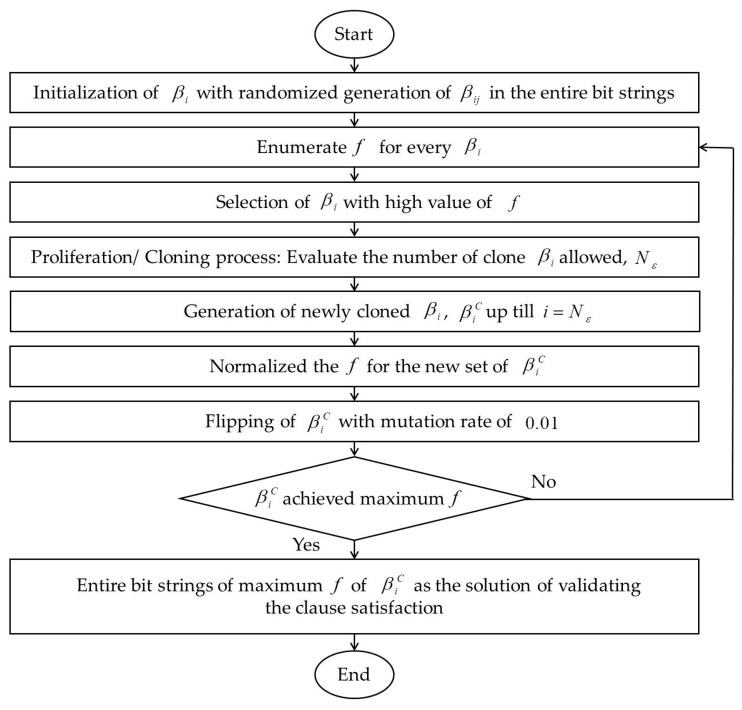
Summary of Clonal Selection Algorithm (CSA).

Finally, the $f_{\beta_{i}}$ of mature population will be computed and we will choose the best β as the candidate cell to be kept in the memory cell. The solution will be selected if $f_{\beta_{i}^{C}}=NC$. On the other hand, the process will repeat from stages 2 to 5 if $f_{\beta_{i}^{C}}\neq NC$. Figure 2 shows the summary of all steps involved in CSA.

## 5. 3-Satisfiability Based Reverse Analysis Method

Logic mining is a process that utilizes logic programming to extract information from a data set. In this regard, this section will explain how the logic mining tool named 3-Satisfiability-based Reverse Analysis Method (3-SATRA) is implemented in our DHNN3-SATCSA model to extract the relationship of AERA entries. Consider n attributes of the data sets $\left(S_{1},S_{2},S_{3},\ldots ,S_{n}\right)$, where $S_{i}\in \left\{1,-1\right\}$. Note that all binary representations must be represented in terms of bipolar states. Since this paper investigates $\varphi_{3-\mathrm{SAT}}$, the arrangement of each $A_{m}$ consists of $S_{i}$, $S_{j}$, $S_{k}$ where i≠j≠k. Note that NC is the number of clauses in $\varphi_{3-\mathrm{SAT}}$. For $A_{m}$ that leads $\varphi_{{}_{3-\mathrm{SAT}}}^{\mathrm{learn}}=1$, we assign

(17) $$A_{m}=\left(S_{i}^{\max\left[n\;\left(S_{i}\right)\right]}\vee S_{j}^{\max\left[n\;\left(S_{j}\right)\right]}\vee S_{k}^{\max\left[n\;\left(S_{i}\right)\right]}\right)$$

Note that $\max\left[n\left(S_{i}\right)\right]$ signifies the highest frequency of $S_{i}$. In this case, each $S_{i}$ is given as follows:

(18) $$S_{i}=\left\{ \begin{array}{l} S_{i}\;,\;S_{i}=1\\ {\bar{S}}_{i}\;,\;S_{i}=-1 \end{array}\right.$$

By using the obtained $A_{m}$, we can formulate $\varphi_{{}_{3-\mathrm{SAT}}}^{\mathrm{best}}$:

(19) $$\varphi_{3-\mathrm{SAT}}^{\mathrm{best}}=\vee_{m=1}^{NC}A_{m}$$

**Figure 3:**
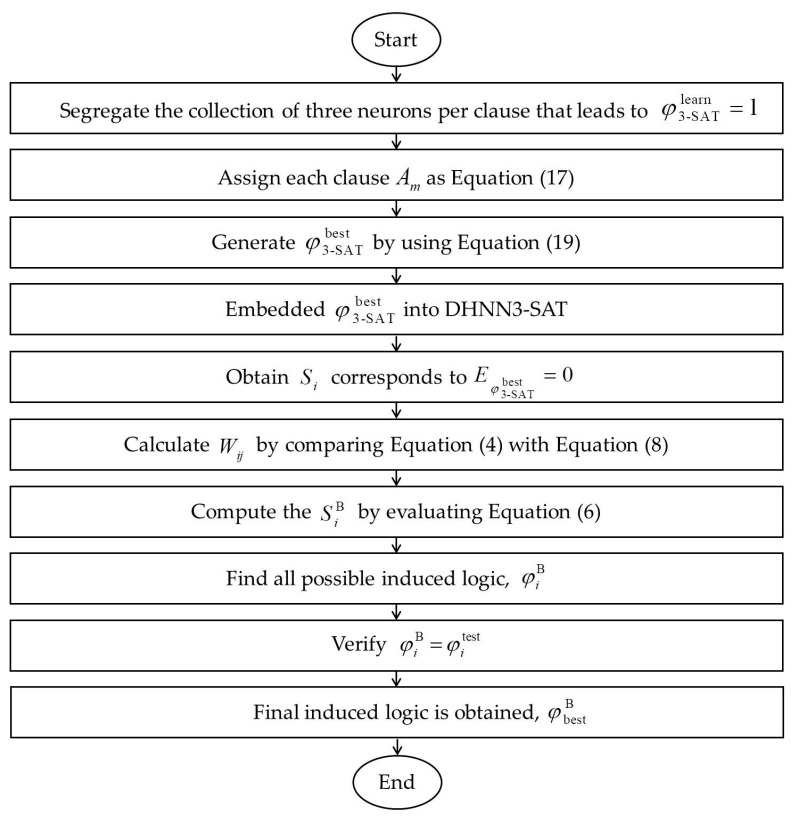
Implementation of 3-Satisfiability Reverse Analysis (3-SATRA) in the Discrete Hopfield Neural Network (DHNN).

For example, we will choose $A_{1}=\left(S_{1}\vee {\bar{S}}_{2}\vee {\bar{S}}_{3}\right)$ if $S_{1}^{\max\left[n\;\left(S_{1}\right)\right]}=S_{1}$, $S_{2}^{\max\left[n\;\left(S_{2}\right)\right]}={\bar{S}}_{2}$ and $S_{3}^{\max\left[n\;\left(S_{3}\right)\right]}={\bar{S}}_{3}$. Next, $\varphi_{{}_{3-\mathrm{SAT}}}^{\mathrm{best}}$ will be embedded into DHNN. Henceforth, we will obtain the states of $S_{i}$ that correspond to $E_{\varphi_{{}_{3-\mathrm{SAT}}}^{\mathrm{best}}}=0$. By comparing Equation (4) with Equation (8), the corresponding $W_{ij}$ will be obtained. During the testing phase, the induced states, $S_{i}^{B}$, will be obtained by using Equation (6). Subsequently, the induced logic, $\varphi_{i}^{B}$ will be constructed based on the rule given in Equation (2). Finally, the chosen induced logic is obtained based on $\varphi_{i}^{B}=\varphi_{i}^{\mathrm{test}}$ (testing data). Figure 3 demonstrates how 3-SATRA was implemented in the DHNN model. In this paper, we will represent each neuron with entries of AERA.

## 6. Experimental Setup

A standard procedure among ANN practitioners is to investigate the proposed model with other comparative studies. Therefore, the simulation process is divided into three sections. Firstly, the performance of DHNN3-SATCSA is analyzed by using simulated data sets. In this case, the ability of CSA in the learning phase of the proposed model will be compared with other existing methods [58,60]. Secondly, several benchmark data sets will be implemented into DHNN3-SATCSA. The comparison of the retrieval properties of DHNN3-SATCSA will be also evaluated based on $\varphi_{i}^{B}=\varphi_{i}^{\mathrm{test}}$. The third section presents the implementation of AERA into the proposed model. All real-life data sets that were converted to bipolar representation and information extraction will be conducted via 3-SATRA and incorporated with DHNN3-SAT models.

In the first section, DHNN with linearized initial neuron states might result in the biasedness of the retrieval state because the network simply memorizes the final state without producing a new state [61]. Therefore, possible positive and negative biases can be reduced by generating all the neuron states randomly as in Equation (20):

(20) $$S_{i}\left(t\right)=\left\{ \begin{array}{cc} 1\;, & rand\left[0,1\right]<0.5\\ -1\;, & \mathrm{otherwise} \end{array}\right.$$

where $S_{i}$ is defined as in Equation (3). The simulated data set will be initiated by generating randomized clauses and literals for each $\varphi_{3-\mathrm{SAT}}$. A similar approach has been implemented in several studies such as [19,60] in generating the initial neuron states. It is worth mentioning that all simulations will be measured against existing methods by evaluating appropriate performance evaluation metrics. By quoting several relevant studies that implemented such experimentations [35,49,62], the proposed performance metrics in this experiment are mean absolute error (MAE), sum of square error (SSE), global minima ratio (ω), accuracy in percentage (α) and computational time in SI unit of second (CT). According to [63], MAE computes the average absolute error of the fitness during the learning phase in our proposed model. The formulation of MAE is as follows:

(21) $$MAE=\sum_{i=1}^{n}\frac{1}{n}\left|\tau -\upsilon \right|$$

where τ and υ are the total number of clauses and the number of satisfied clauses in $\varphi_{3-\mathrm{SAT}}$ respectively. In relation to Equation (21), the accumulation of errors in each model can be also effectively evaluated by using SSE. The formulation of SSE is described by the following equation:

(22) $$SSE=\sum_{i=1}^{n}{\left(\upsilon -\tau \right)}^{2}$$

On the other hand, we examine the final neuron states of the proposed model via ω.

(23) $$\omega =\frac{1}{ab}\sum_{i}^{n}N_{L_{\varphi_{3-\mathrm{SAT}}}}$$

According to [52], if the final neuron states of the proposed model is $E_{\varphi_{3-\mathrm{SAT}}}\to 0$, the model will prone to ω→1. Hence, the best model will attain the lowest value of MAE and SSE with ω→1. Notably, ω→1 indicates $n\left(\left|L_{\varphi_{3-\mathrm{SAT}}}^{\min}-L_{\varphi_{3-\mathrm{SAT}}}\right|\leq \lambda \right)\to ab$ where a and b are number of trials and neuron combinations, respectively. In another development, we utilize the value of α and CT to investigate the effectiveness and efficiency of 3-SATRA in the testing phase of DHNN3-SATCSA. We describe two formulations:

(24) $$\alpha =\frac{\varphi_{i}^{B}}{N_{\varphi_{i}^{\mathrm{test}}}}\times 100\%$$

(25) CT=LearningTime (s)+RetrievalTime (s)

Note that α→100 if $n\left(\varphi_{i}^{\mathrm{test}}=\varphi_{i}^{B}\right)\to G$, where G in our case is depicted by 40% of instances in a data set. In practice, the best model requires α→100 and the minimum value of CT. Learning time and retrieval time are denoted as total time executed by DHNN3-SAT models in the learning phase and retrieval phase respectively. Table 2 and Table 3 discuss the parameters involved in hybrid Hopfield Neural Network with Exhaustive Search (DHNN3-SATES) and hybrid model with Clonal Selection Algorithm (DHNN3-SATCSA) respectively.

**Figure 4:**
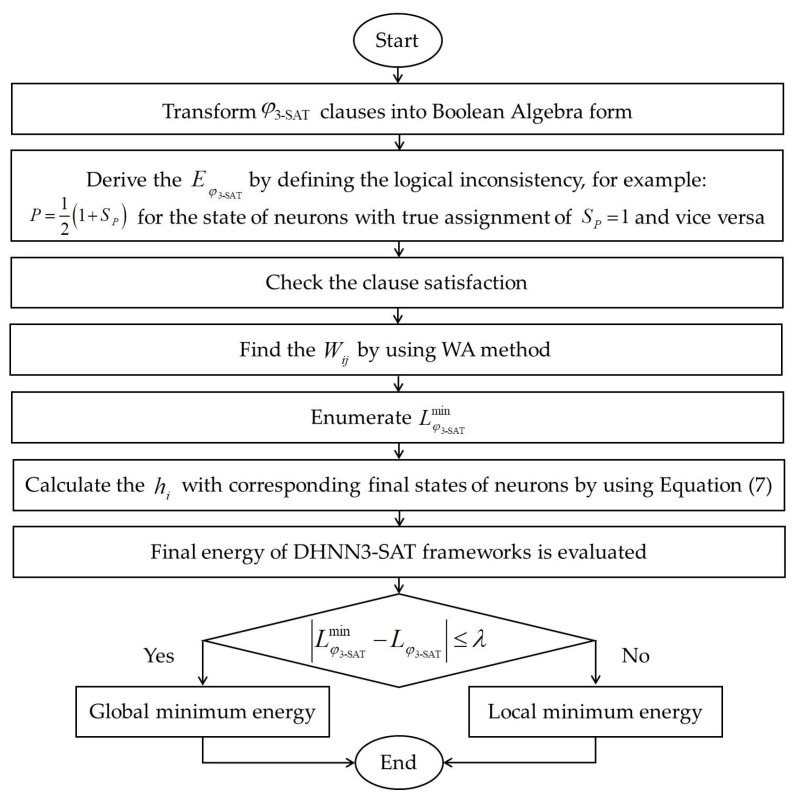
The implementation of DHNN3-SAT models in a simulated data set.

The choice of β is important as a large population size requires a large searching space of the solutions, which may increase the computational cost. On the other hand, a small β can lead to local minima solutions. According to [64], we should choose β=100 as it is repeated to achieve a good result. The general implementation of the proposed model in a simulated data set can be summarized in Figure 4. 3-SATRA is implemented to show the level of connectedness between $W_{ij}$ and neurons. Overall, simulated and real-life data sets will be implemented into DHNN3-SATCSA. The computational simulation for both data sets was conducted on Dev C++ Version 5.11 for Windows 7 in 2GB RAM with Intel Core I3. As for the simulated data set, the Dev C++ program will generate the initial bipolar data randomly. Throughout the simulations, the same device is being used to avoid any biases. On the whole, all simulations are utilized with different number of neurons (NN), which is within the bound of not exceeding the threshold time of 24 h [35]. Note that the proposed model will randomly select nine attributes for the real-life data set as well as their arrangements in $\varphi_{3-\mathrm{SAT}}$ logical rule.

## 7. Results and Discussion

### 7.1. Simulated Data Set

**Figure 5:**
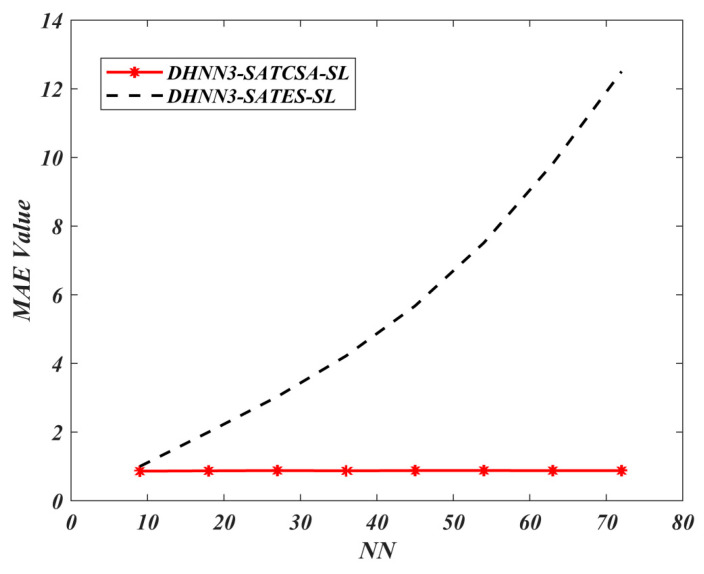
Mean absolute error (MAE) value of DHNN3-SAT models.

**Figure 6:**
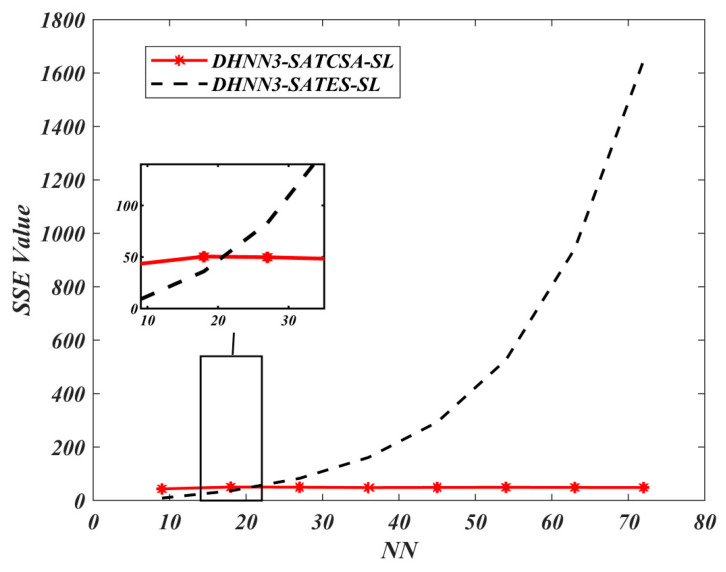
Sum of square error (SSE) value of DHNN3-SAT models.

**Figure 7:**
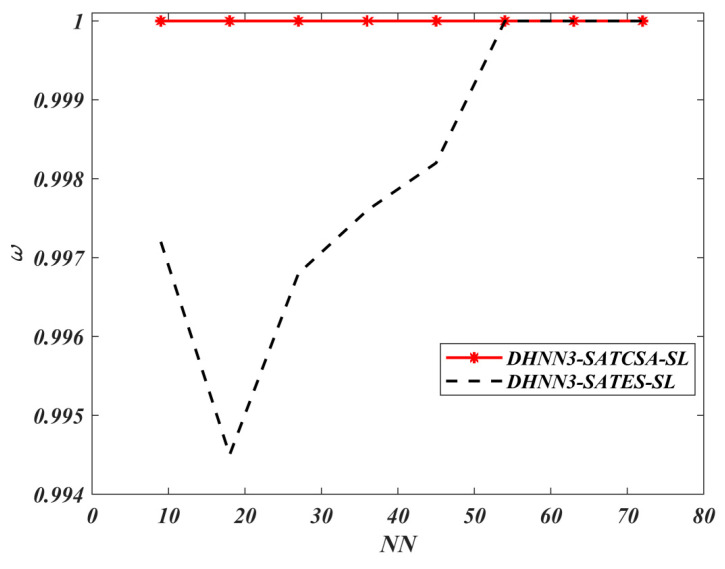
ω value of DHNN3-SAT models.

The first section of the experiment was carried out by using simulated data. This section evaluates the performance of CSA as the learning rule in the DHNN model in comparison with ES. The findings of simulated data set for both models are presented as follows:

According to Figure 5 and Figure 6, DHNN3-SATCSA accumulated fewer errors compared to DHNN3-SATES due to CSA’s ability to learn and train the network effectively. However, ES incorporates random search which makes the complexity of the learning phase increase. As illustrated in Figure 7, DHNN3-SATCSA achieved a consistent value of ω=1, from NN=9 to NN=72 whereas DHNN3-SATES only gained a better value of ω after processing 62.5% of the total NN. ES projected unnecessary projection due to the “trial-and-error” feature that does not aid the proposed model to improve the solutions. CSA can manage a large number of constraints compared to ES. CSA made this possible because CSA showcased the ability of β in fighting the pathogens and improving the affinity values in the entire bit strings to help DHNN3-SATCSA search for ideal solutions. In this experiment, we did not consider α because the value of ω corresponds to the number of global minimum energy achieved by DHNN3-SAT models. Hence, the value of ω is adequate to represent the effectiveness of the retrieval phase of both models. The main distinction between these models with [47] is the formulation of fitness function. The cost function in [47] is $E_{\varphi_{3-\mathrm{SAT}}}\neq 0$ because the structure of $\varphi_{3-\mathrm{SAT}}$ is not satisfiable. CSA reduces the number of iterations because the CSA optimization operator, particularly somatic hypermutation, will allow the solution to attain $E_{\varphi_{3-\mathrm{SAT}}}=0$ faster than ES. In general, CSA will reduce learning time, which will elongate relaxation time within the ideal rate and, we believe, result in less neuron oscillation. It is worth noting that the probability for somatic hypermutation to flip the neurons entirely is approaching to 0. Thus, the chances for the solution to achieve nonimproving fitness will reduce drastically compared to conventional ES. The Wan Abdullah method is chosen because this method is reported to contribute less neuron oscillation as compared to other methods such as Hebbian learning [65]. Uncontrollable neuron oscillation via other methods such as Hebbian learning will lead to more local minimum energy or $\left|L_{\varphi_{3-\mathrm{SAT}}}^{\min}-L_{\varphi_{3-\mathrm{SAT}}}\right|>\lambda$. This comparison is vital to validate the learning capabilities of CSA. The limitation of DHNN3-SATCSA is the use of bipolar neuron states instead of another neuron representation (ternary), $S_{i}\in \left\{1,-1,0\right\}$. Ternary representation can provide more analysis since it has another vector of 0 which indicates no response or meaningless result. In another perspective, the proposed model only considers satisfiable SAT logic. Other SAT representations such as MAX*k*-SAT [18] require major restructuring, especially in terms logical redundancy. Furthermore, this experiment only employs a nonrestricted learning environment where the CSA and ES will iterate until $E_{\varphi_{3-\mathrm{SAT}}}=0$. Finally, this work only embeds $\varphi_{3-\mathrm{SAT}}$ in terms of CNF form. According to [66], CNF representation is more compatible to the WA method compared to Disjunctive Normal Form (DNF) representation.

### 7.2. Benchmark Data Sets

For the second part of the experiment, the simulation is carried out over a set of four widely used benchmark real-life data sets [67] listed in Table 4. Note that this section evaluates the performance of DHNN-3SAT models in doing real life data sets. A benchmark data set is reported in this paper because these structured data sets are validation performances of the DHNN-3SAT models.

All attributes (consisting of nine literals for each data set) listed in Table 4 will be embedded into 3-SATRA using implementation in Figure 3. We choose data set from the different disciplines because each data set has different clustering behaviour. The objective of each DHNN3-SAT model in this section is to induce the best $\varphi_{i}^{B}$ that classifies the outcome of the data sets. In general, the choice of outcome for each data set is given as follows:

1. BDMC: Client will subscribe a term deposit where 1 and −1 signify nonsubscription and subscription respectively.
2. CCDP: Response to default payment of credit card customers where 1 and −1 signifies nonpaymaster and paymaster respectively.
3. DRDD: Signs of diabetic retinopathy where 1 and −1 signifies the sign exist and nonexist respectively.
4. FLST: Customers interest where 1 and −1 signifies the show of noninterest and interest towards the product respectively.

In this section, we only evaluate the performance of the induced $\varphi_{i}^{B}$ and disregard the result from learning error. The instances of the data sets will be divided into $\varphi_{i}^{\mathrm{learn}}$(60%) and $\varphi_{i}^{\mathrm{test}}$(40%) which follows the procedure of the logic-mining model proposed by Kho [35]. We found that more capacity for learned data (than the proposed proportion) will result in data overfitting. Thus, the best DHNN-3SAT model is measured based on the highest value of α.

**Figure 8:**
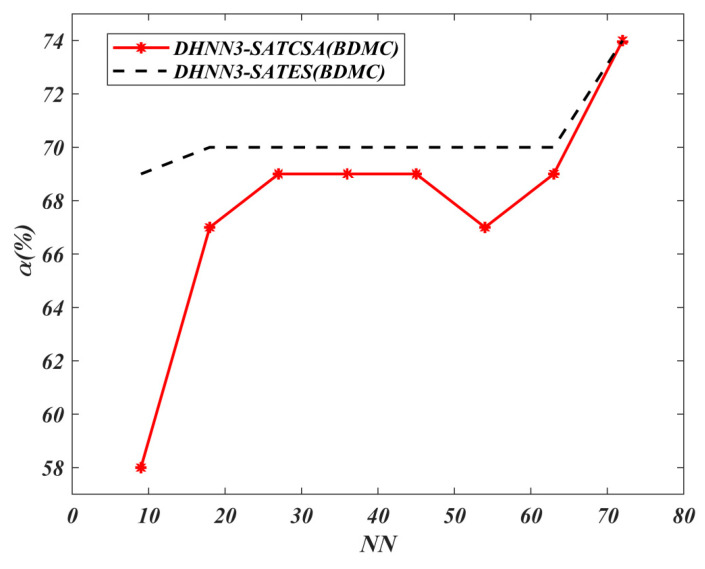
α (%) value of DHNN3-SAT models in the BDMC data set.

**Figure 9:**
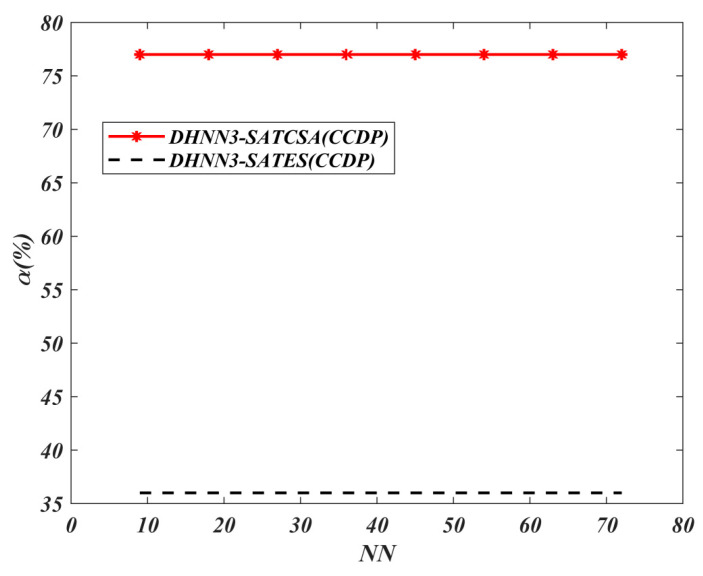
α (%) value of DHNN3-SAT models in the CCDP data set.

**Figure 10:**
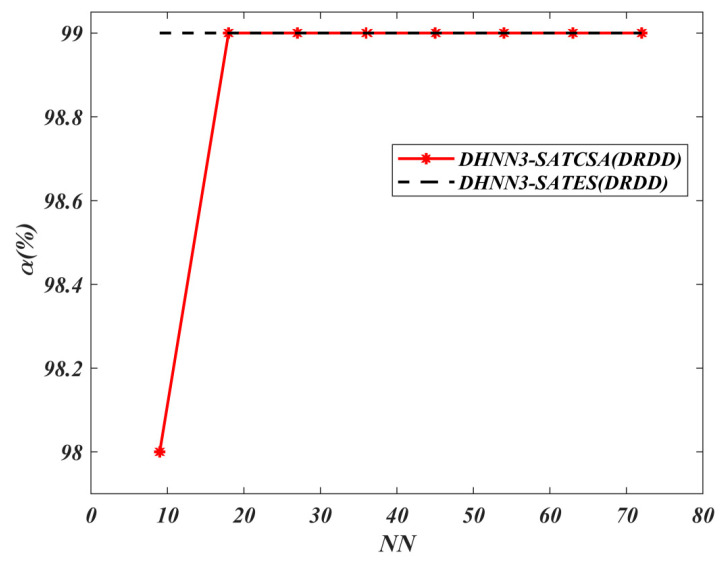
α (%) value of DHNN3-SAT models in the DRDD data set.

**Figure 11:**
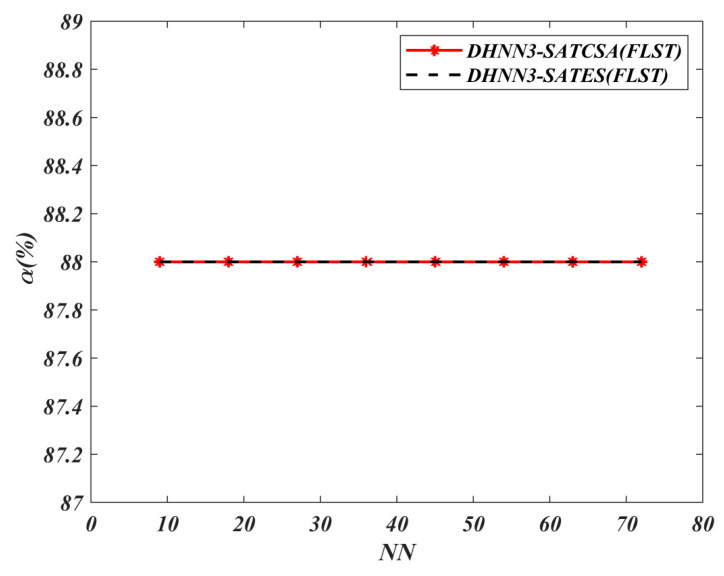
α (%) value of DHNN3-SAT models in the FLST data set.

The value of α for all models is shown in Figure 8, Figure 9, Figure 10 and Figure 11. A higher value of α indicates the optimality of the model in retrieving $\varphi_{i}^{B}$. The results of the analyses discussed in Figure 8, Figure 9, Figure 10 and Figure 11 are all based on the assumption that $\varphi_{i}^{B}$ for all data sets had achieved ω=1. According to Figure 8, both models demonstrate the similar maximum value of α=74% for NN=72 in the BDMC data set. Despite the similar value of α for both models at NN=72, DHNN-3SATES reported high learning error compared to DHNN-3SATCSA. The small value of α for DHNN-3SATCSA at 10≤NN≤60 is due to overfitting the solution during the retrieval phase of DHNN-3SAT. As seen in Figure 9, the overall trend of α is distinct where the proposed model achieved consistent value of 77% and 36% for all NN respectively. In Figure 10 and Figure 11, the proposed DHNN-3SATCSA is reported to not be effective when the NN is small, although the α reached the maximum value at NN≥15. Despite a similar value of α for both models in Figure 11, DHNN-3SATCSA achieved similar α in lower learning error. As observed in Figure 8, Figure 9, Figure 10 and Figure 11, the proposed DHNN-3SATCSA in 3-SATRA exhibits competitive performance with respect to the learning error and α. The innovation of DHNN-3SATCSA lies in the solution diversity of β that prevents CSA from getting trapped in local minima energy. In this case, a promising β will be improved via hypermutation strategy during the learning phase of DHNN. In contrast, DHNN-3SATES has no optimization layer and in most cases will contribute to suboptimal $\varphi_{i}^{B}$ (see Figure 9). We expect that DHNN-3SATES will exceed the threshold computational time when NN>88 due to the structural limitation of ES. Hence, we can further agree that generally DHNN3-SATCSA is a better model in terms of α and the capability of its mechanism to employ different sizes and natures of real-life data sets. We can further improve the retrieval property of DHNN-3SATCSA by implementing a mutation operator such as in [19].

Table 5 extends the experiment by comparing benchmark data sets with other existing methods which comprises conventional statistical methods such as decision tree (DT), naïve Bayes (NB), support vector machine (SVM). The work of [68] utilized BDMC to predict the successful direct marketing campaign that ensures customers subscribing to a term deposit plan by using DT analysis. Our proposed model achieved better α with differences of 12.73%. On the other hand, the α attained by [69] for CCDP is excessively lower than our proposed model. [69] applied an NB classifier to provide information for risk management of handling customers with credit risks. In [70], this work applies SVM analysis with a confusion matrix to accentuate feature selection and classification. However, the α gained for the SVM method is 25.3% lower than DHNN3-SATCSA. As for FLST, there is no comparable recent work that utilizes this data set.

Note that the proposed model does not consider the effect of attribute permutation. This straightforward $\varphi_{i}^{\mathrm{learn}}$ implementation helps us to effectively determine attributes in the induced logic $\varphi_{i}^{B}$ whenever we convert it to other logic programming form. It is worth mentioning that this simulation only considers attributes that lead to $\varphi_{3-\mathrm{SAT}}^{\mathrm{learn}}=1$, because the proposed model aims to minimize the $E_{\varphi_{3-\mathrm{SAT}}}=0$. Since there are no redundant attributes in 3SATRA, the satisfiability aspect of $\varphi_{i}^{B}$ can be guaranteed. In this case, the structure of $\varphi_{i}^{\mathrm{learn}}$ should be modified into nonsatisfiability logic such as maximum satisfiability [58]. By that standard, we expected DHNN-3SATCSA will outperform DHNN-3SATES if $E_{\varphi_{3-\mathrm{SAT}}}\neq 0$ is considered in 3-SATRA. In addition, the proposed DHNN-3SAT model does not consider noise function such as in the work of [19]. Thus, the result from this section is important as the $\varphi_{i}^{B}$ can be easily analysed by the practitioners as compared to relying entirely on error analysis. Through our findings of simulated and benchmark data sets, we further experiment the competency of DHNN3-SATCSA in entrenching AERA by analysing several performance evaluation metrics.

### 7.3. Amazon Employees Resources Access Data Set

#### 7.3.1. Performance of DHNN3-SAT in Learning and Testing Phase

From the previous section, we can conclude that the proposed model is suitable to implement in AERA. Therefore, this section investigates the behaviour of DHNN3-SATCSA analysing AERA to help benefit Amazon.com Inc. Both models utilized the 3-SATRA method. However, our main contribution is to investigate CSA capability to enhance the learning mechanism of DHNN. This is to ensure an optimal learning environment. Relative to the experiment, the key findings of the attained $\varphi_{\mathrm{best}}^{B}$ will also be presented in this section.

**Figure 12:**
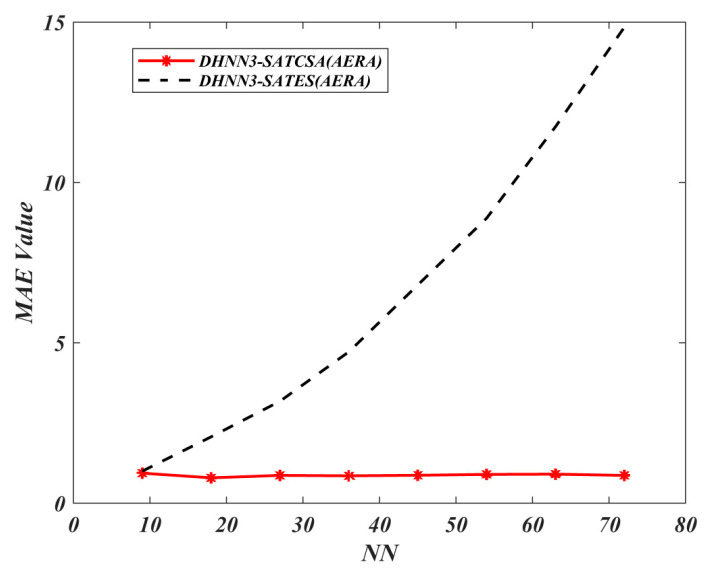
MAE value of DHNN3-SAT models.

**Figure 13:**
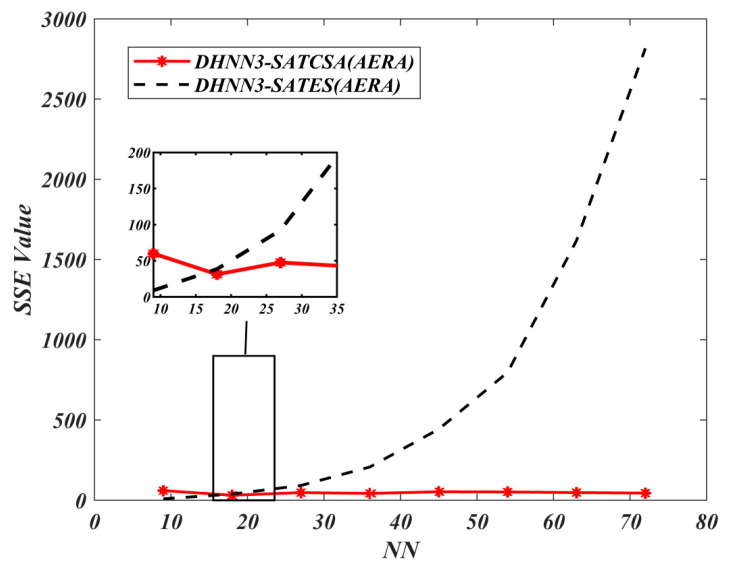
SSE value of DHNN3-SAT models.

**Figure 14:**
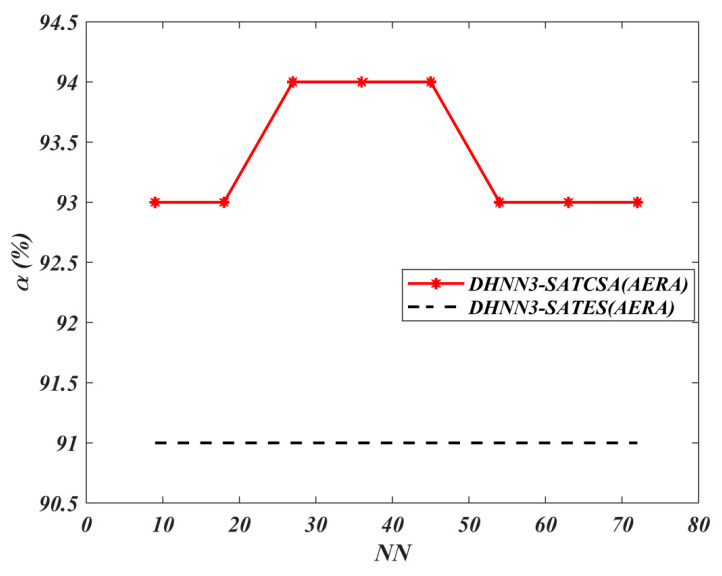
α(%) value of DHNN3-SAT models.

**Figure 15:**
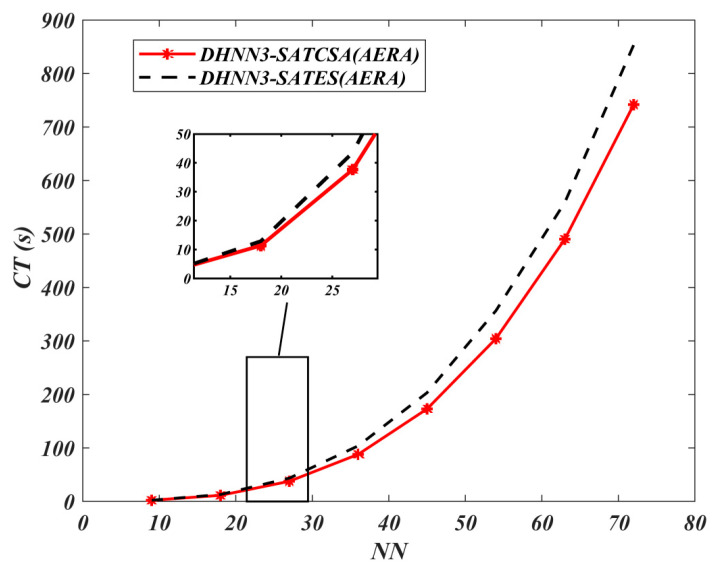
CT value of DHNN3-SAT models.

In Figure 12 and Figure 13, both the MAE and SSE of DHNN3-SATCSA attained consistent value of errors, approaching to 0, whereas, for DHNN3-SATES, the errors are gradually increasing. Particularly for DHNN3-SATCSA, less accumulation of errors is due to the CSA mechanism improving the quality of solutions in order to attain $E_{\varphi_{3-\mathrm{SAT}}}=0$. However, ES generated larger value of errors because the ES mechanism is only effective with low NN. We illustrate the capability of the retrieval properties of DHNN3-SAT models based on Figure 14 and Figure 15. Overall, the value of α obtained by DHNN3-SATCSA is relatively higher by at most 3% compared to DHNN3-SATES. Conversely, we also compare the α obtained by other existing work such as [4] that also utilized AERA with conventional statistical methods such as LR, GB and RF. Due to the imbalanced entries of AERA, the work also mentioned their efforts of constructing a prediction model by trying out single models on categorical data. Subsequently, it was improved by introducing various modified methods of decision trees to finally get the desired α. A summary of α achieved by DHNN3-SATCSA with all comparative methods are shown in Table 6. From NN=9 up to NN=36, the CT recorded for both models have a similar rate. However, from NN=45 onwards, DHNN3-SATES required more CT. The apparent reason of why DHNN3-SATES needed more time compared to DHNN3-SATCSA is because the ES mechanism leads to a property of entire bit strings of logical rule collapsing when any of the clauses is not satisfied, thus more iterations are required to produce a plausible solution. That is unlike CSA’s ability to minimize iterations in the completion of learning process due to its optimization operator [64].

#### 7.3.2. Key Findings of $\varphi_{\mathrm{best}}^{B}$

Equation (26) shows the attained $\varphi_{i}^{B}$ at the highest α($\varphi_{\mathrm{best}}^{B}$) by DHNN3-SATCSA. The generated $\varphi_{\mathrm{best}}^{B}$ will help Amazon.com Inc. in identifying insignificant factors to improve the human resources management. Table 7 shows the details of AERA utilized in this experiment.

(26) $$\varphi_{\mathrm{best}}^{B}=\left(P\vee \bar{Q}\vee R\right)\wedge \left(\bar{S}\vee T\vee \bar{U}\right)\wedge \left(\bar{V}\vee W\vee \bar{X}\right)$$

Equation (26) gives information of which attributes carry a trivial role in Amazon.com Inc. employees resources applications. We recognized the negation of literals in Equation (26) as a factor that does not affect the problem faced by Amazon.com Inc. For example, $\bar{Q}$ indicates a manager’s unnecessary role to grant resources application. This is believed to add more pointless human administration to solve the employees’ complications regarding their resources. In addition, attributes like P will influence the application process as the availability of resources is required to be known for sufficient needs of all employees. P also provides resources information to other departments like the operations and maintenance departments to manage defective equipment and appliances. R and $\bar{S}$ are correlated. However, it shows clearly different major levels of management, such as top-level, middle-level and first-level are crucial in deciding which resources are first in line. The example of roles related to R and $\bar{S}$ are engineers and retailers respectively, thus Equation (26). We can conclude that engineers should be prioritized first compared to retailers as Amazon.com Inc. emerges as a well-known tech giant. Furthermore, Amazon.com Inc. should prioritize T to decide which departments are more vital and need new resources to accomplish their tasks in the company. Amazon.com Inc. have to underline certain standards to maintain the quality of work from certain departments that hold a greater role in the norm business of Amazon.com Inc. Consequently, factors like $\bar{U}$ clearly show the insignificant need of considering the business title of an employee in order to grant or revoke their employees resources applications. Top management of Amazon.com Inc. could improve business personnel in a value-added business to other employees in their business duties. The attributes of $\bar{V}$ and W are related to one another, however, the difference is the specification of an employee role. $\bar{V}$ is the extended version or additional role given to an employee, by referring to the attained $\varphi_{\mathrm{best}}^{B}$. We can deduce that Amazon.com Inc. should only consider employees’ main role in the company to prioritize the resources applications. Attribute $\bar{X}$ is only essential when Amazon.com Inc. recollects which role is open to vacancy and does not affect much in resources management of Amazon.com Inc.

In line with the no free lunch theorem [72], it is impractical to propose a specific algorithm or model which claims to solve all real-life applications. Thus, new developments on improving metaheuristics and optimization model are continuously needed to handle particular optimization tasks. This work focused on DHNN3-SATCSA transforming AERA into 3-SAT logic representation with 3-SATRA to generate optimum $\varphi_{\mathrm{best}}^{B}$ to extract information from AERA. On the other hand, [73] reports that the CSA mechanism computational time may take longer because the number of affinity evaluations is increasing as the population of β increases. Nonetheless, the $\varphi_{\mathrm{best}}^{B}$ attained from DHNN3-SATCSA may help provide Amazon.com Inc. an alternative model to predict resources applications of the future set of employees. Furthermore, DHNN3-SATCSA could be tested by implementing other types of optimization problems from other companies, such as Walmart’s efforts to reduce food waste through distribution processes or Ikea’s attempts to scale up their system of product fault detection. The implementation of DHNN3-SATCSA will provide beneficial information to a company that wishes to know which factors are more significant than others and which could lead to better control and management of their production.

## 8. Conclusions

In conclusion, we believe the findings of this study will broaden fundamental optimization methods, such as statistical methods or conventional evolutionary algorithms. In this experiment, the incorporation of 3-SAT in DHNN was crucial to exhibit the relationship and behaviour of AERA symbolically. In addition, 3-SATRA was developed in this study to extract information from AERA, despite its large size with imbalanced entries. Subsequently, 3-SATRA is vital to generate induced logics which displayed insignificant factors in AERA that lead to the problem faced by Amazon.com Inc. Also, the construction of our modified DHNN3-SAT model integrated with modified CSA was revealed to be useful to improve the traditional learning phase of DHNN. In addition, we demonstrated the competency of our hybrid DHNN model of DHNN3-SATCSA by entrenching three different data sets: simulated, benchmark and AERA, in comparison with other existing methods. The comparative investigation was executed by employing various performance evaluation metrics. The findings showed DHNN3-SATCSA outperformed other existing methods. In order to construct a possible model that can cater to all optimization tasks, further improvement of the proposed model could be done to improve the performance and mechanism of the model by implementing a mutation feature in the testing phase of DHNN. Therefore, the exploration of the testing phase in DHNN is worthy of attention, alongside future research addressing the variability of implementing other algorithms to enhance the mechanism of modified DHNN models.

## Figures and Tables

**Table 1:** Example of cases for the 3-Satisfiability (3-SAT) logical rule, $\varphi_{3-\mathrm{SAT}}$.

Case	$\varphi_{3-\mathrm{SAT}}\;\mathrm{Instances}$	Outcome
1	(P,Q,R,S,T,U,V,W,X)=(1,1,1,−1,−1,−1,1,1,−1)	Satisfiable $\left(\varphi_{3-\mathrm{SAT}}\;=1\right)$
2	(P,Q,R,S,T,U,V,W,X)=(1,−1,−1,1,1,−1,1,1,1)	Unsatisfiable $\left(\varphi_{3-\mathrm{SAT}}\;=-1\right)$
3	(P,Q,R,S,T,U,V,W,X)=(−1,1,1,1,−1,−1,−1,1,1)	Full consistency $\left(\varphi_{3-\mathrm{SAT}}\;=1\right)$
4	(P,Q,R,S,T,U,V,W,X)=(1,−1,−1,−1,1,1,1,−1,−1)	Full inconsistency $\left(\varphi_{3-\mathrm{SAT}}\;=-1\right)$

**Table 2:** List of parameters in DHNN3-SATES [52].

Parameter	Parameter Value/Remarks
a	100
b	100
λ	0.001
CT	24 h
NN	9≤NN≤72
Selection Rate	0.1
Number of Strings	100
Type of Selection	Random

**Table 3:** List of parameters in DHNN3-SATCSA.

Parameter	Parameter Value/Remarks
n(β)	100
γ	200
θ	0.01
λ	0.001
CT	24 h
NN	9≤NN≤72
Type of Selection	Roulette Wheel Selection [59]
Learning Method	WA Method [20]

**Table 4:** List of benchmark data sets information.

Benchmark Data Sets/Field	Attributes	Instances	Sources
Bank Direct Marketing Campaign (BDMC)/Marketing	P: Age	45,211	UCI Machine Learning Repository
	Q: Job		
	R: Credit card status		
	S: Housing loan		
	T: Personal loan		
	U: Last contact day of the month		
	V: Last contact duration		
	W: Number of days passed by after the client was last contacted from a previous campaign		
	X: Number of contacts performed before this campaign		
Credit Card Default Payment (CCDP)/Finance	P: Amount of limit balance	3000	UCI Machine Learning Repository
	Q: Education		
	R: Marital status		
	S: History of repayment status in Month I		
	T: History of repayment status in Month II		
	U: Amount of bill statement in Month I		
	V: Amount of bill statement in Month II		
	W: Amount of previous payment in Month I		
	X: Amount of previous payment in Month II		
Diabetic Retinopathy Debrecen Disease (DRDD)/Health	P: Result of quality assessment	1151	UCI Machine Learning Repository
	Q: Result of pre-screening		
	R: Features detection I		
	S: Features detection II		
	T: Features detection for exudates I		
	U: Features detection for exudates II		
	V: Affected patient condition according to the Euclidean Distance (center of the macula and the center of the optic disc)		
	W: Diameter of the optic disc		
	X: Result of the AM/FM- based classification		
Facebook Live Sellers in Thailand (FLST)/Marketing	P: Status type	7050	UCI Machine Learning Repository
	Q: Number of comments		
	R: Number of shared post		
	S: Number of likes		
	T: Number of reaction; Love emoticon		
	U: Number of reaction; Wow emoticon		
	V: Number of reaction; “Haha” emoji		
	W: Number of reaction; “Sad” emoji		
	X: Number of reaction; “Angry” emoji		

**Table 5:** α of DHNN3-SATCSA in comparison with other existing methods.

Data Set	DHNN3-SATCSA	ES	α/Method
BDMC	74%	74%	61.27%/DT [68]
CCDP	77%	36%	66.32%/NB [69]
DRDD	99%	99%	73.7%/SVM [70]
FLST	88%	88%	-

**Table 6:** α of DHNN3-SATCSA model in comparison with other existing methods.

Method	α
DHNN3-SATCSA	94%
ES [65]	91%
LR [1]	87.21%
RF [1]	85.58%
GB [1]	85.14%

**Table 7:** List of information on Amazon Employees Resources Access (AERA) 2010–2011 data set.

Attributes	Example	Instances/Sources
P: An ID for each resources	Types of resources (computer, laptops, software)	
Q: Manager employee ID	Supervised or not supervised employee	
R: Company role up category ID 1	US Data Analyst	
S: Company role up category ID 2	US Manufacturing	
T: Company role department	Manufacturing	32,769/Kaggle Machine Learning and Data Science Community [71]
U: Company role business title	Junior Data Analyst, Senior Manufacturing Staff	
V: Company role family extended description	Security Data Analyst, Product fault detection manufacturing staff	
W: Company role family description	Security Data Analyst	
X: Company role code (unique to each role)	Data Analyst	
